# Heterogeneity of dose distribution in normal tissues in case of radiopharmaceutical therapy with alpha-emitting radionuclides

**DOI:** 10.1007/s00411-022-01000-5

**Published:** 2022-10-14

**Authors:** Wei Bo Li, Céline Bouvier-Capely, Clarita Saldarriaga Vargas, Michelle Andersson, Balázs Madas

**Affiliations:** 1 Helmholtz Zentrum München-German Research Center for Environmental Health (GmbH), Institute of Radiation Medicine, Neuherberg, Germany; 2 Institut de Radioprotection et Sûreté Nucléaire (IRSN), PSE-SANTE/SESANE/LRSI, Fontenay-aux-Roses, France; 3 Radiation Protection Dosimetry and Calibrations, Belgian Nuclear Research Centre (SCK CEN), Mol, Belgium; 4 In Vivo Cellular and Molecular Imaging Laboratory, Vrije Universiteit Brussel, Brussels, Belgium; 5 Medical Physics Department, Jules Bordet Institute, Université Libre de Bruxelles, Brussels, Belgium; 6 Environmental Physics Department, Centre for Energy Research, Budapest, Hungary

**Keywords:** Dose heterogeneity, Radiopharmaceutical therapy, Alpha-emitter, Normal tissue, bone marrow, Patient dosimetry

## Abstract

Heterogeneity of dose distribution has been shown at different spatial scales in diagnostic nuclear medicine. In cancer treatment using new radiopharmaceuticals with alpha-particle emitters, it has shown an extensive degree of dose heterogeneity affecting both tumour control and toxicity of organs at risk. This review aims to provide an overview of generalized internal dosimetry in nuclear medicine and highlight the need of consideration of the dose heterogeneity within organs at risk. The current methods used for patient dosimetry in radiopharmaceutical therapy are summarized. Bio-distribution and dose heterogeneities of alpha-particle emitting pharmaceutical ^223^Ra (Xofigo) within bone tissues are presented as an example. In line with the strategical research agendas of the Multidisciplinary European Low Dose Initiative (MELODI) and the European Radiation Dosimetry Group (EURADOS), future research direction of pharmacokinetic modelling and dosimetry in patient radiopharmaceutical therapy are recommended.

## Introduction

In nuclear medicine, radiopharmaceuticals are administered into the human body for diagnostic and therapeutic purposes (Bolch et al. 2009; ICRU 2021). Radiopharmaceuticals are designed to target one specific organ or tissue in the body, to bind to malignant cells and/or destroy cancers. In this process, however, other healthy organs and tissues are concurrently irradiated. Because of the varying local physiology of the human body, the biochemical properties of the radiopharmaceuticals, and the type of radiation emitted, the distribution of radionuclides and energy deposition within an organ or tissue might be subject to a large heterogeneity (Adelstein 1993). In addition, the variation of anatomical structures in the human body, the distances among different organs, and the substructures sub-organ and even subcellular levels will contribute further dose heterogeneity. This must be taken into consideration in radiation transport simulations within the human body (ICRU 2021). In radiopharmaceutical therapy, the use of radionuclides emitting alpha particles is particularly useful because, due to the short range of these particles in tissue, the energy deposition is localised close to tumours, increasing tumour-specific cell killing and sparing normal tissue. While in diagnostic procedures absorbed doses are typically low (< 20 mGy) for most organs, this is not so for therapeutic applications where absorbed doses can range from a few gray up to a hundred or more gray (Gy) (EANM 2017; Stokke et al. 2017). At these dose levels, a mixture of stochastic effects and tissue reactions is expected (Aerts et al. 2021). Therefore, appropriate assessment of radiation dose heterogeneity in tumours and normal tissues is crucial to assess the benefit-risk balance of the treatment and to optimize the therapeutic outcome.

As novel radiopharmaceuticals are emerging for therapeutic use, there is a need for patient-specific dosimetry both in tumour and normal organ tissues (ICRU 2002; EC 2014; ICRP 2019). Furthermore, doses to sub-regions of specific organs, for example, the bone marrow or the endosteum in bone tissues, must be known to establish dose–response relationships required in dosimetry-based prescriptions (Sgouros et al. 2020; St James et al. 2021). To develop any generalized dose assessment formalism in therapeutic applications, some difficulties need to be resolved, such as: tumour dose cannot be directly assessed; patient-specific images and anatomy must be taken into account for individual dose calculation; sub-organs and sub-regions within tumours must be considered. Furthermore, the anti-tumour efficacy and organs-at-risk toxicity need to be predicted that therapeutic index can be estimated (ICRP 2019).

In 2013, ^223^Ra dichloride (Xofigo) was approved by the US Food and Drug Administration (FDA) for the treatment of bone metastases in patients with metastatic castration-resistant prostate cancer (mCRPC) (EMA 2018). Parker and Sartor (2013) demonstrated in a phase III study that the treatment with Xofigo extends the overall survival time of patients versus placebo by 3.6 months. As Xofigo is an alpha-particle emitting bone-seeking radionuclide, the dose heterogeneity in bone marrow (which can be considered as a bone sub-tissue) plays an important role in the bone dose–effect relation for bone toxicity during the Xofigo therapy.

Radiopharmaceutical uptake is often not uniform within an organ or tissue. In the case of Xofigo treatment, the short range of the alpha particles emitted by ^223^Ra and its progeny leads to a highly heterogeneous dose distribution in bone tissue and the average absorbed dose over bone marrow will be not predictive of the potential biological effects, since the local absorbed dose to bone marrow will be greater than the average absorbed dose due to the bone-seeking property of the radionuclide ^223^Ra. Consequently, bone marrow dose–effect modelling for alpha-emitter requires the consideration of the heterogeneity of the dose distribution on a microscale (Sgouros et al. 2020). This review mainly focusses on the heterogeneity of radionuclide and dose distribution in tissues and organs, such as bone marrow, resulting from a treatment by Xofigo, to exemplify organ dose calculations for similar radiopharmaceuticals. Finally, in line with the strategic research agendas (SRAs) of the Multidisciplinary European Low Dose Initiative (MELODI) and the European Radiation Dosimetry Group (EURADOS), relevant research priorities related to radiopharmaceutical therapy are identified.

## Heterogeneity of radiopharmaceutical bio-distribution in nuclear medicine

A challenge in radiopharmaceutical dosimetry is to accurately characterize the spatially heterogeneous distribution of any radiopharmaceutical and its radioactive progeny at the scale of radiosensitive tissues and their substructures. This heterogeneous spatial distribution leads to an inhomogeneous energy deposition. As a first challenge, in the case of radiopharmaceutical therapy with alpha-emitters, in which the decay energy of alpha-particles is sufficient to break chemical bonds, the parent radionuclide and the decay progeny may readily dissociate from the targeting agent (Kunos et al. 2021). The free radionuclides may thus relocate and result in an increased radiation dose to untargeted tissues. A second challenge is that the distribution of radiopharmaceuticals is time-dependent not only due to physical decay, but also because the bio-distribution of any radiopharmaceutical is driven by individual patient pharmacokinetics. Therefore, knowledge of the therapeutic radionuclide bio-distribution in patients at different time points after administration of the radiopharmaceutical, the so-called time–activity curve, is required for accurate organ dose estimation.

In the future, the so-called theranostic approach is anticipated to play a major role in the management and health care of patients with cancer in nuclear medicine (Eberlein et al. 2017; Lassmann et al. 2021; ICRU 2021). This approach includes labelling of carrier molecules with a radionuclide suitable for imaging, which allows to localize the radiopharmaceutical in tumour and normal tissues, followed by administration of the companion therapeutic agent (same carrier, but labelled with a different radionuclide) that irradiates the tumour with a therapeutic dose. The imaging stage allows to estimate the uptake of the radiopharmaceutical in normal and tumour tissues to predict expected radiation doses and adjust the therapeutic activity that will be administered to the patient in the treatment stage. This strategy may lead to enhanced therapeutic efficacy, alleviate adverse events, and finally result in improved patient outcome.

In clinical practice, imaging is often used to determine the 3D or 2D activity distribution in the body of the patient, which is necessary to estimate the dose to tumours and organs at risk. Radionuclides that decay via positron emission can be imaged with positron emission tomography (PET), whereas those that emit photons during decay can be imaged using single-photon emission computed tomography (SPECT). Clinical PET typically achieves a spatial resolution of 4–6 mm full-width at half maximum (FWHM), while SPECT imaging has a resolution of 7.5–15 mm FWHM (St James et al. 2021). In addition, accurate volume contouring is performed using computed tomography (CT) or magnetic resonance imaging (MRI). These combined imaging techniques make it possible to calculate absorbed dose to tumour and normal tissues based on actual patient anatomy. However, dosimetry is not only needed for estimation of absorbed doses in organs but also for assessment of related biological effects. The main issue in radiopharmaceutical therapy, in particular with alpha-emitters, is that organ toxicity is usually determined by radiopharmaceutical distribution on a microscopic scale that may not be resolved with the clinical imaging techniques mentioned above (Sgouros et al. 2020; St James et al. 2021). In particular, estimation of mean absorbed dose in whole organs and tumours is not enough to accurately predict the biological outcome of alpha-particle therapies. Thus, the MIRD committee (Sgouros et al. 2010) and other investigators (McDevitt et al. 1998; Hobbs et al. 2011) have recommended small-scale dosimetry.

Imaging could also be useful in preclinical studies to explore the relationship between absorbed dose and any biological effects due to radiation exposure, in particular for alpha emitters for which bio-distribution information at the tissue, cellular, and subcellular level is required. However, on a small scale, the resolving power of clinical imaging detectors is not sufficient and specific instruments need to be developed to predict and explain tumour response and healthy organ toxicities. Several studies have been published on the use of digital autoradiography technology employing a new generation of position-sensitive charged-particle imagers for ex vivo imaging of alpha-emitters in tissue sections of normal and tumour tissues (Frost et al. 2015; Miller et al. 2015). For example, an alpha-camera can visualize and quantify important differences and temporal changes in activity distributions of alpha-emitting bio-conjugates both in normal tissues and tumours (Bäck and Jacobsson 2010). The good spatial resolution (about 25 µm FWHM for alpha particles) of this type of camera allows to compare activity uptake in micro-metastases versus isolated tumour cells. With this instrument it was shown that the activity uptake per cell is lower in large micro-metastases than in isolated tumour cells, which can be useful information to interpret the outcome of preclinical therapeutic efficacy studies (Chouin et al. 2013). These studies demonstrated that the alpha-camera can be used to quantify both spatial and temporal activity distributions in sacrificed animals and, thus, can be implemented in small-scale dosimetry and microdosimetry studies.

Miller and colleagues (Miller et al. 2014; Miller 2018) developed a similar iQID camera which is applicable to a broad range of ionizing radiation types including alpha-particles. This camera was used for quantitative imaging of ^211^At distribution in cryosections of murine and canine tissue samples, with a spatial resolution of about 20 µm FWHM (Miller et al. 2015). Due to its low alpha-particle background, activity distribution measurement was performed at mBq µg^−1^ levels. All these studies demonstrated that high-resolution autoradiography can be used to quantify both spatial and temporal activity distributions in sacrificed animals and, thus, it can provide information relevant for small-scale dosimetry and microdosimetry studies. Moreover, these ex vivo high-resolution imaging techniques are not limited to preclinical experiments. It can be expected that they could be applied to the analysis of tumour material excised from patients undergoing surgery (Chouin et al. 2013).

## Fundamentals of radiation dosimetry in nuclear medicine

Organ absorbed dose for radiopharmaceuticals used in nuclear medicine can generally be calculated by the MIRD schema (MIRD 1968; Loevinger and Berman 1975). A generalized dosimetry formalism has recently been published by MIRD and ICRP (Bolch et al. 2009; ICRP 2016). Assessment of organ absorbed dose can be described by Eq. (1) in a time-independent way follows:

1 $$D\left( {r_{T} ,T_{D} } \right) = \mathop \sum \limits_{{r_{S} }} \tilde{A}\left( {r_{S} ,T_{D} } \right)S\left( {r_{T} \leftarrow r_{S} } \right),$$

where $D\left({r}_{T},{T}_{D}\right)$ is the absorbed dose in target tissue *r*_T_; $\widetilde{A}\left({r}_{S},{T}_{D}\right)$ is the time-integrated activity (TIA) in source tissue *r*_S_, i.e. the activity integrated over time period *T*_*D*_; $S({r}_{T}\leftarrow {r}_{S})$ is the mean absorbed dose rate to target tissue *r*_*T*_ per unit activity present in source tissue *r*_*S*_.

The time-dependent formulation is described by Eq. (2):

2 $$d\left( {r_{T} ,T_{D} } \right) = \mathop \sum \limits_{{r_{S} }} \mathop \smallint \limits_{0}^{{T_{D} }} a\left( {r_{S} ,t} \right)S\left( {r_{T} \leftarrow r_{S} ,t} \right)dt,$$

where $d\left({r}_{T},{T}_{D}\right)$ is the absorbed dose coefficient in target tissue *r*_T_; $a\left({r}_{S},t\right)$ is the fraction of the administered activity in the source tissue *r*_S_ at time *t* post administration; $S({r}_{T}\leftarrow {r}_{S},t)$ is the mean absorbed dose rate to target tissue *r*_*T*_ per unit activity present in source tissue *r*_*S*_ at time *t*, called the *S* coefficient (or *S* value), which is given by Eq. (3).

3 $$\begin{gathered} S\left( {r_{T} \leftarrow r_{S} ,t} \right) = \frac{1}{{M\left( {r_{T} ,t} \right)}}\mathop \sum \limits_{i} E_{i} Y_{i} \phi \left( {r_{T} \leftarrow r_{S} ,E_{i} ,t} \right) \hfill \\ \quad \quad \quad \quad \quad \, = \frac{1}{{M\left( {r_{T} ,t} \right)}}\mathop \sum \limits_{i} {\Delta }_{i} \phi \left( {r_{T} \leftarrow r_{S} ,E_{i} ,t} \right) \hfill \\ \quad \quad \quad \quad \quad \, = \mathop \sum \limits_{i} {\Delta }_{i} {\Phi }\left( {r_{T} \leftarrow r_{S} ,E_{i} ,t} \right) , \hfill \\ \end{gathered}$$

where *E*_*i*_ is the mean (or individual) energy of the *i*th nuclear transition; *Y*_*i*_ is the yield of the *i*th nuclear transition per nuclear transformation; $\phi ({r}_{T}\leftarrow {r}_{S},{E}_{i},t)$ is the absorbed fraction (AF), which is defined as the fraction of radiation energy *E*_*i*_ emitted by source tissue *r*_S_ at time *t* that is absorbed in target tissue *r*_T_; *M*_T_ is the mass of the target tissue *r*_T_ in the reference individual; and $\Phi \left({r}_{T}\leftarrow {r}_{S},{E}_{i},t\right)$ is the specific absorbed fraction (SAF) value at time *t*, which is defined as the ratio of the AF and the target mass *M(r*_*T*_*,t)* in different organs of the administered radiopharmaceutical at time *t*. The *S* coefficients are calculated for specific reference anatomical models (so-called phantoms) using radiation transport simulations typically based on the Monte Carlo method. Such phantoms mimic the geometry and the elemental composition of tissues in the human body, typically to the organ level and sometimes to the level of main sub-organ tissue regions or subcellular regions.

Besides the energy deposition in the organs and tissues where the decay takes place, radiation also deposits energy in proximate organs and tissues. Therefore, in addition to assess the biokinetics, the cross-fire dose from radionuclides located in distant tissues must also be assessed. To do so, anatomic information of the human body is required. Previously, this has been modelled by mathematical phantoms, while now this is being modelled by computational voxel phantoms, non-uniform rational basis spline (NURBS) phantoms and polygon-mesh phantoms. A comprehensive review of the evolution of computational phantoms for radiation dosimetry has been reported by Xu and Eckerman (2009) and (Bolch et al. 2010). After implementing such human phantoms in Monte Carlo codes, the *S* coefficients for organs can be calculated. In the development of anthropomorphic models used for organ dose calculation in nuclear medicine, one can see changes of the *S* coefficients from the first-generation stylized phantoms towards the current polygon-mesh phantom (Stabin and Xu 2014).

## Dosimetry for radiopharmaceutical therapy

The MIRD dosimetry schema is a general formalism for calculation of mean absorbed dose from internally distributed radionuclides over spatial dimensions ranging from subcellular to organ levels (Loevinger and Berman 1975; Loevinger et al. 1991; Bolch et al. 2009). This schema provides the foundation for radiopharmaceutical therapy dosimetry. However, it is not appropriate for toxicity and anti-tumour efficacy evaluation relevant to radiopharmaceutical therapy (Sgouros et al. 2020). Several assumptions in the dose calculation schema confine its application, for example, the standardised source–target geometries; the uniform distribution of radionuclides in the source organs and the calculation of averaged absorbed dose to organs (ICRU 2002). Nonetheless, in the course of the development of new radiopharmaceuticals, such as ^177^Lu-PSMA-targeting ligands and Xofigo, the MIRD schema was implemented for radiopharmaceutical therapy (ICRU 2021). It is noted that the EC Directive 2013/59/Euratom (EC 2014) states in article 56 that exposures of target volumes in nuclear medicine treatments shall be individually planned. Exposure should appropriately be verified taking into account that doses to non-target volumes and tissues shall be as low as reasonably achievable and consistent with the intended radiotherapeutic purpose of the exposure (Konijnenberg et al. 2021). This article 56 motivated to review some individual patient dosimetry methods for the new emerging radiopharmaceuticals in therapy by adopting concepts used in external beam radiation therapy (EBRT) and, in the same time, by taking into account the current MIRD/ICRP schema (Bolch et al. 2009) and ICRU methodology (ICRU 2002, 1979).

The voxel-level based dosimetry strategy has been proposed for patient-specific dose assessment in tumours and organs at risk (Bolch et al. 1999; Sgouros et al. 2008, 2010, 2020; Dewaraja et al. 2012; Ljungberg and Gleisner 2015; Ljungberg and Sjögreen Gleisner 2016; Ljungberg and Sjogreen Gleisner 2018). Recently, Sgouros et al. (2021) regarded the dosimetry of all radiopharmaceutical therapy agents as a challenge and demanded that the absorbed dose should be independent of the physicist performing the dose assessment, the software used, and the institution where the imaging is performed. Such standardization is essential for multi-centre trials. The general strategy for voxel-level based dosimetry is as follows: (i) acquire serial quantitative PET/CT or SPECT/CT scans; (ii) deformably register the CT scans and associated activity images; (iii) segment organs, tumours, or other regions of interest; (iv) perform a dose calculation for each scan, which yields a dose-rate map at each time point; and finally (v) model and integrate the dose rate as a function of time within each voxel and region to obtain a final dose value for each voxel (Graves and Hobbs 2021).

The scheme of voxel-level absorbed dose calculation is similar to the MIRD absorbed dose method (Eq. 4) (Sgouros et al. 2020):

4 $$\begin{gathered} D\left( {r_{T} } \right) = \mathop \sum \limits_{i} \tilde{A}\left( {x_{i} ,y_{i} ,z_{i} } \right) \cdot K\left( {r_{i} } \right) \hfill \\ \tilde{A}\left( {x_{i} ,y_{i} ,z_{i} } \right) = \int_{0}^{\infty } {A\left( {x_{i} ,y_{i} ,z_{i} ,t} \right)dt} , \hfill \\ \end{gathered}$$

where $D\left({r}_{T}\right)$ is the absorbed dose in the target voxel *r*_T_; $A({x}_{i},{y}_{i},{z}_{i},t)$ is the 3D matrix representing the spatial radioactivity distribution at time point *t* in voxel element r $({x}_{i},{y}_{i},{z}_{i})$; $\widetilde{A}({x}_{i},{y}_{i},{z}_{i})$ is the time-integrated activity (TIA) in each voxel element; and $K({r}_{i})$ is the source-to-target distance-dependent absorbed dose per unit TIA, referred to as the dose point kernel.

This dose point kernel can be calculated for charged and uncharged particles and, as well, for homogenous and non-homogenous media, where the Monte Carlo simulation method must be applied. Generally, three main approaches have been used for the calculation of absorbed doses for radiopharmaceutical therapy (ICRU 2021; Sgouros and Hobbs 2014): (1) the direct Monte Carlo method; (2) the dose point kernel method; and (3) the voxel-wise *S* coefficients method. These three principal approaches of absorbed dose calculation show an inherent relation, where the Monte Carlo simulation method plays a fundamental role.

## Direct Monte Carlo method

Monte Carlo (MC) radiation transport simulation is the fundamental approach for absorbed dose calculation in radiopharmaceutical therapy. In biological material, energy transfer of an alpha-particle occurs through excitations and ionizations and is described by the stopping power which is defined as the average energy loss per unit distance along its path. However, the energy lost by an alpha-particle transfers to secondary radiation, like electrons and photons which penetrate to distances further away from the alpha-particle tracks. The energy of an electron is mainly transferred to matter through interaction of the electric field of moving electrons with that of electrons bound in the medium. This interaction, mainly by the inelastic collision, leads to electronic excitations and ionizations, and to slowed-down electrons with residual energies less than 10 eV, which are locally absorbed. Photons interact with molecules in three main processes: photoelectric effect, Compton scattering, and electron–positron pair production. The cross sections for these processes in liquid water and other materials are well calculated and available to quantify the energy deposition in biological materials (Attix 1986). With the cross sections of radiation transport in biological material, MC techniques are applied to simulate the radiation interaction with matter with a computer event-by-event by randomly sampling the processes from the ratios of their cross sections to total cross sections (Andreo 1991).

The MC method is independent to, but needed by the other two approaches mentioned above. The MC method is applicable to (i) inhomogeneous media such as, lungs and bone and soft tissues; (ii) complex geometries; and (iii) conditions where radiation and charged-particle equilibrium are not fulfilled. Furthermore, in radiopharmaceutical therapy, the MC method can be applied to the complete range of targets and non-targets including various levels of heterogeneity, from the whole body of patients, to organ and sub-tissue levels, and further to cellular and molecule levels including the DNA. Further advantage of applying the MC method in radiopharmaceutical therapy is the integration of physical, physicochemical, chemical, and biological effect modelling, which can be used to investigate the factors underlying the biological effects in tumours and normal tissues upon radiotherapy. However, some limitations of the MC method hamper its use in practical clinical radiopharmaceutical therapy, such as the demanding CPU simulation time required and the large uncertainties of cross sections for low-energy particles. The first limitation may be solved by applying GPU or large CPU clusters in the MC simulation.

## Dose point kernel method

The dose point kernel method is a very simple model to use and that can be performed with different MC codes in case of a homogenous medium. In the dose point kernel method, the dose deposition from an isotropic point source is calculated as a function of the distance from the source. In radiopharmaceutical therapy, the calculation of the dose kernel is usually performed using radiation transport simulations based on the MC method, by scoring the energy deposition in concentric spherical shells around a point source in a homogeneous medium (St James et al. 2021). This method was first used for mono-energetic electron sources in water (Berger 1973). The dose point kernel method is now used for different tissues and for different radiation types.

Taking account of tissue heterogeneities, the dose point kernel can be scaled linearly as a function of the effective density of the medium, which is proportional to the relative mass density of the medium and water (Cross 1968). Because the dose point kernel is a non-stochastic quantity, it is less useful for the situation of stochastic fluctuations of energy deposition in a subcellular target volume. In this case, the direct MC method should be applied. Furthermore, the dose point kernel should not be used for nonhomogeneous media, especially for alpha and beta-particles. In this case, the direct MC method is again more suitable (ICRU 2021).

## Voxel-wise *S* coefficients method

The voxel-wise *S* coefficients method (these coefficients are also called individualized *S* coefficients in ICRU report 67 and radionuclide *S* coefficients in ICRU report 96) was developed as an extension of the MIRD formalism. Actually, this method uses the *S* coefficient as defined in the framework of the MIRD schema (St James et al. 2021; ICRU 2021). As shown in Eq. (3), the *S* coefficient represents the mean absorbed dose to a defined target region *r*_T_ per nuclear transformation of any radionuclide of interest deposited uniformly within a defined source region *r*_S_. The *S* coefficient is computed for a specific radionuclide and a specific source-target geometry, taking into account the distance of the source and target regions, and the composition of the corresponding materials and their densities. In case of alpha-emitters with decay products, *S* coefficients for the decay products must be calculated separately taking the biokinetic distribution of each progeny in the source regions into account. As represented in Eq. (3), the *S* coefficient is generally derived from the *SAF* or rather AF, which was calculated for mono-energetic radiation of different types, mostly photons, electrons, and alpha-particles, by performing MC radiation transport simulations with a defined source-target geometry. Typically, this geometry includes voxel phantoms in different scales: whole organ, sub-tissue, clinically acquired voxel image, and individual cell (ICRU 2021). There are several computer programmes available that can be used for the calculation of *S* coefficients by applying transformation techniques (for example, fast Fourier transformation) or the density correction method. In these programmes, *S* coefficients are calculated in advance for different radionuclides and specific voxel geometries and then stored in a database for practical use. However, the transformation technique might not be appropriate for regions with substantial heterogeneity, such as bone marrow in skeletal sites (St James et al. 2021). In such cases, the direct MC simulation method should be used for calculation of specific voxel-wise *S* coefficients.

## Computational models for sub-tissue bone marrow

**Fig. 1:**
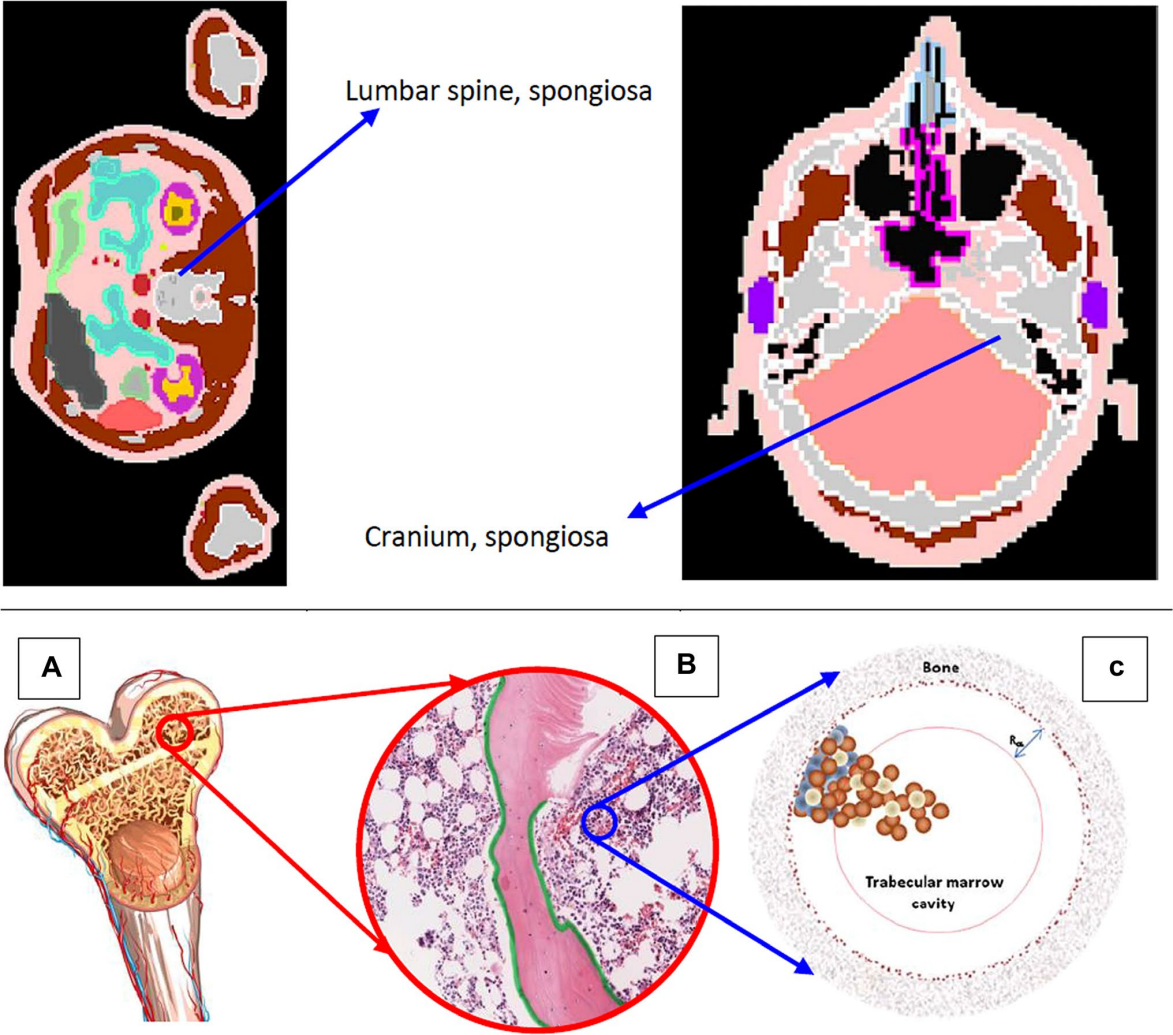
Upper panel: ICRP voxel phantom of spongiosa in lumbar spine and cranium (ICRP 2009). Lower panel: from bone to bone marrow phantom (Hobbs et al. 2012), **A** Structure of upper arm bone (humeri) which shows the spongiosa, the medullary cavity and the cortical bone; **B** Spongiosa which shows the trabecular bone, red bone marrow, yellow bone marrow and the endosteum; **C** Mathematical phantom model for red bone marrow, which shows the trabecular marrow cavity and the osteoprogenitor cells (blue), hematopoietic stem and progenitor cells (brown), and adipose cells (white). Figures reproduced with permission by ICRP and IOP Publishing

Heterogeneous activity distributions of radiopharmaceuticals can result in heterogeneous distributions of the dose within the tissues. In that case, the MIRD method based on *S* coefficients for whole organs is of limited use, as it does not permit to accurately estimate the dose absorbed locally within the tissue of interest. In this session, we make an overview of the computational phantoms of bone marrow tissues currently available which allow to perform dosimetry for specific regions at sub-tissue level (Fig. 1).

Because of the short range of alpha-particles, typically 50–80 µm in tissue depending on alpha-particle energy, a highly heterogeneous dose distribution results in bone marrow upon alpha-particle emitting radiopharmaceutical therapy. While the mean absorbed dose to the bone marrow is useful to predict the haematological toxicity from antibody and peptide-coupled alpha-particle emitting radiopharmaceuticals (Wahl et al. 2021), it overestimates the potential biological effects for patients due to bone seeking radiopharmaceuticals like Xofigo (Sgouros et al. 2020). Consequently, for red bone marrow dosimetry, a more detailed structure within the bone marrow which includes the bone surface cells should be developed to quantify the heterogeneous dose distribution to the bone marrow cells. Furthermore, this would allow prediction of the biokinetics of any free progeny on a microscopic scale. Bone marrow models that have been developed are reviewed below, and their potential use in the dosimetry of alpha-particle emitting radiopharmaceuticals is explored.

**Fig. 2:**
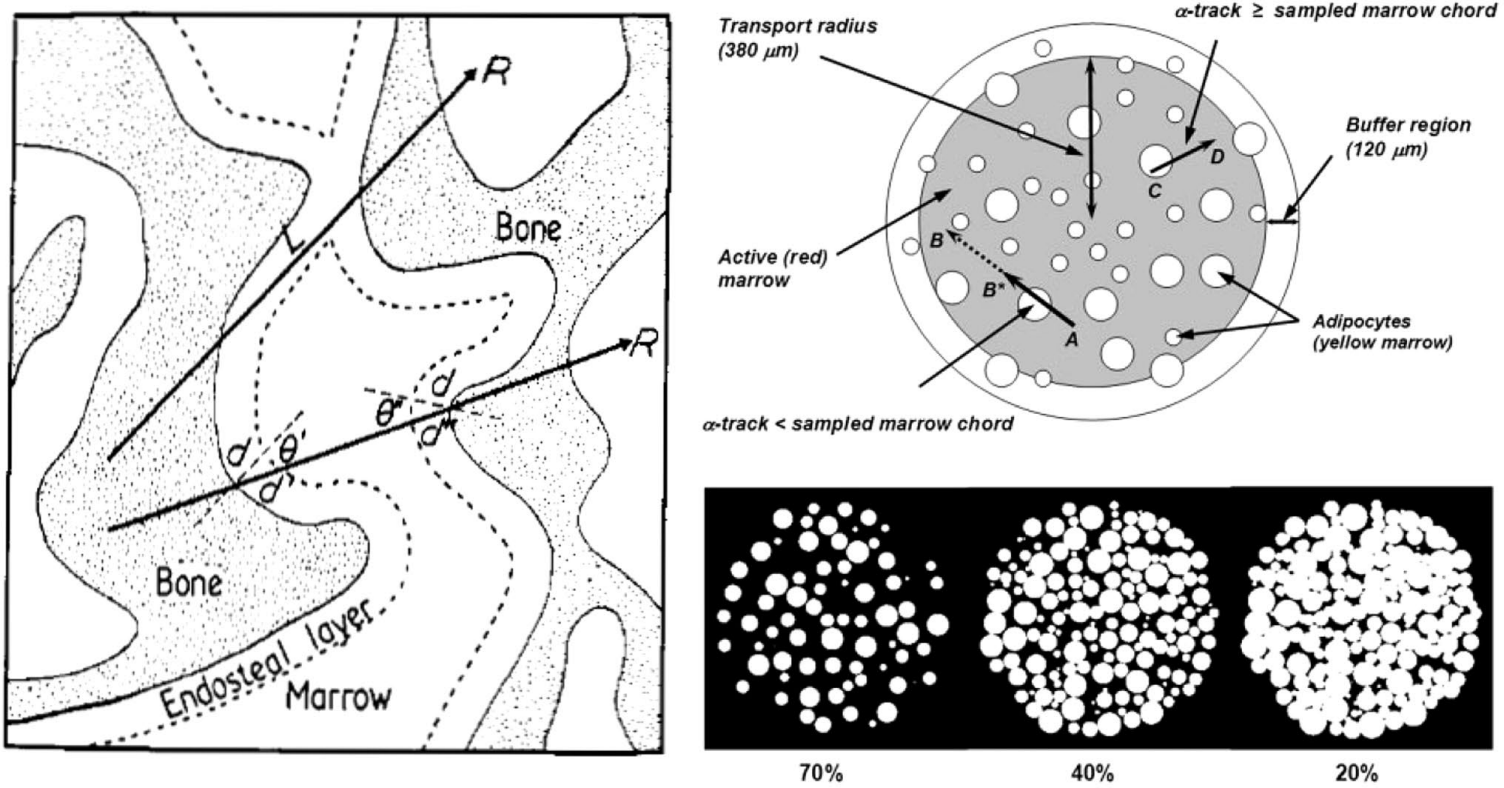
Left: path-length bone marrow model (Whitwell and Spiers 1976; Beddoe et al. 1976). Right: 3D marrow tissues model (Watchman et al. 2005). Upper right pannel not drawn to scale. Figures reproduced with permission by IOP Publishing and SNMMI

Because of the cancellous irregular microstructure of the bone marrow, it is difficult to model the bone anatomic structure in computational dosimetry. Images of the relevant tissue of bone trabecular and marrow cavity cannot be acquired directly by in vivo imaging systems like CT and MR. Therefore, images of excised trabecular spongiosa which were previously acquired ex vivo by micro-CT or MRI microscopy were mostly used for developing skeletal dosimetry models. Spiers and colleagues developed a relatively complete path-length model through trabeculae of the cervical vertebra measured with physical sectioning and automated light microscopy for a 44 year male subject across seven skeletal sites (Whitwell and Spiers 1976; Whitwell 1973; Beddoe et al. 1976) (Fig. 2, left). Unfortunately, in this model the skeletal tissue masses were not reported. Nevertheless, the modelled trabecular bone and marrow cavities were sufficient for voxel-level dose calculations. Taking this 3D microstructure model of trabecular and marrow cavities, which was obtained from a 44 year male, a supplemental 3D spatial model of marrow tissue was inserted within the marrow cavity (Watchman et al. 2005). The marrow tissue model consists of two regions. The first region is the inner sphere (with a radius of 380 µm) of marrow modelling the marrow cellularity (in its composition percentage of 70, 40 and 20%) with red (or active) marrow and a varying fraction of yellow (or adipocyte) marrow within the marrow sphere (Fig. 2, right). The second region is the buffer region, a shell (with a width of 120 µm) around the centre of the marrow cavity (Fig. 2, right). This 1000 µm diameter sphere of the marrow spatial model (not shown in Fig. 2) corresponds to the nominal chord length of the marrow cavity for the investigated 44-year male (Whitwell and Spiers 1976). This combined model can be used for estimating *S* coefficients for red marrow irradiated by alpha-particle-emitting radiopharmaceuticals, such as those labelled with ^211^At, ^223^Ra, ^225^Ac and ^227^Th, distributed in regions within bone.

Later, Jokisch et al. (1998) generated images of trabecular regions within a human thoracic vertebra, which were obtained with a high-field proton nuclear MRI at a field strength of 14.1 T. These images were digitally processed to measure the chord length distributions through both the bone trabeculae and marrow cavities. The distributions yielded a mean trabecular thickness of 201 µm and a mean marrow cavity thickness of 998 µm, which are both qualitatively consistent with those reported in (Whitwell and Spiers 1976).

**Fig. 3:**
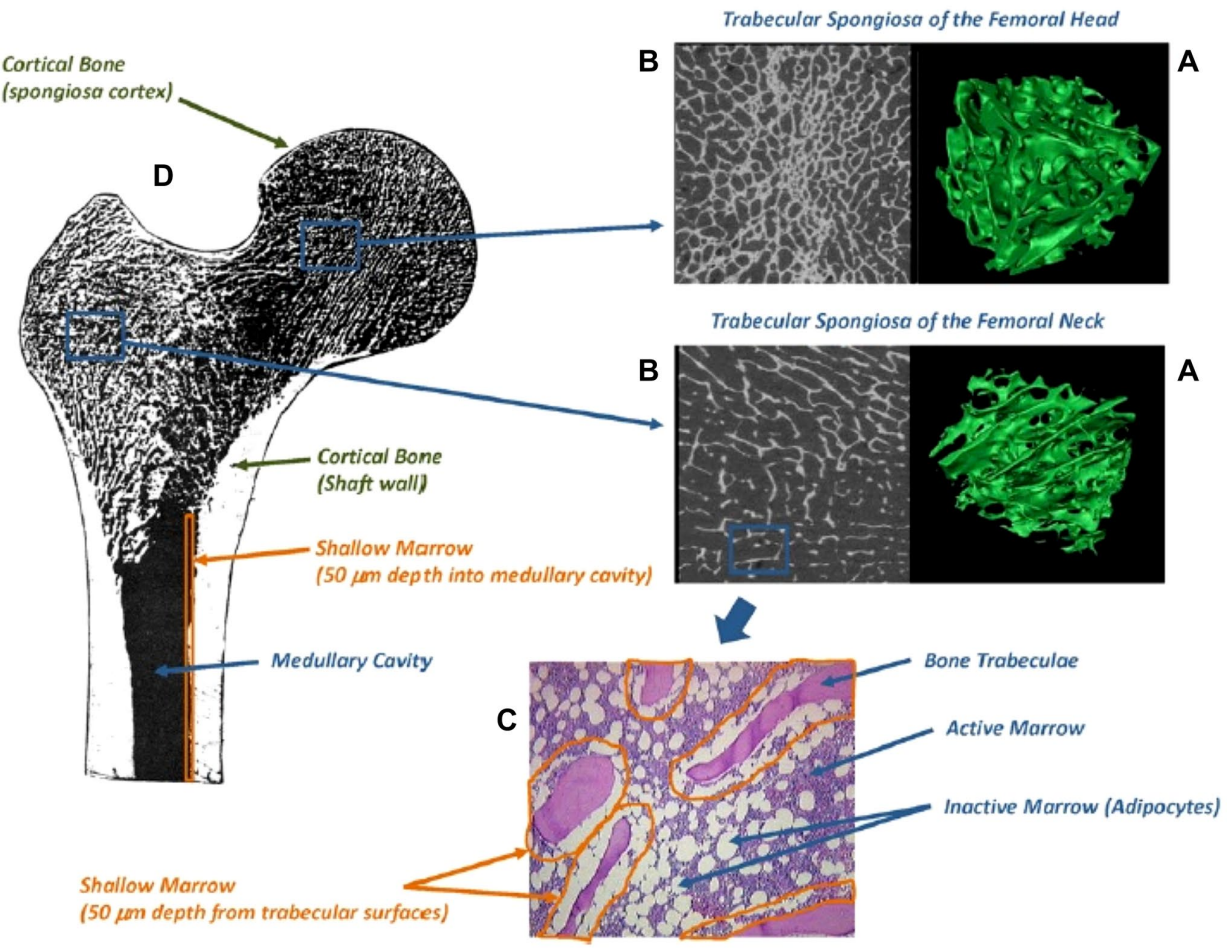
**A** NURBS-type 3D surface model (in green colours) within the trabecular spongiosa regions of the femoral head and neck; **B** 2D micro-CT images of spongiosa regions; **C** shallow marrow (50 mm depth layer) from trabecular surface along the inner cortical surfaces of the medullary cavity of the **D** diaphysis (shaft) (Hough et al. 2011). Figures reproduced with permission by IOP Publishing

More recently, micro-CT imaging was used to investigate cadavers and construct a skeletal dosimetry model with detailed images of trabeculae spongiosa for a 64 year adult male (Shah 2004) and a 66 year adult female (Kielar 2009). In assembling the adult male skeletal model, the cores of marrow intact spongiosa were excised from each collected bone sample. All samples were imaged with a micro-CT system at a 30 μm isotropic voxel size. After image segmentation of spongiosa and cortical bone in ex vivo CT images, multiple image processing steps were applied to the micro-CT images of the spongiosa cores to assess both the marrow volume fraction and trabecular bone volume fraction of spongiosa by skeletal site (Hough et al. 2011). This adult male bone model has been integrated into the ICRP reference adult male for calculating *S* coefficients in bone tissue and red bone marrow (Hough et al. 2011). The micro-CT images of the trabecular spongiosa acquired from the 64-year adult male was first converted to a NURBS-type surface model, shown in green colours for femoral head and neck (Fig. 3). In Fig. 3, the cancellous images beside the green colour images are the 2D micro-CT images seen from the rightmost columns. Furthermore, the shallow marrow at a depth of 50 mm (where osteoprogenitor cells are present) from the trabecular surface is also shown Fig. 3. This NURBS-type spongiosa model was voxelized and further converted to a voxel phantom to be used for bone marrow dosimetry calculations with MC codes. Application of the female bone marrow model (Kielar 2009) in the ICRP reference adult female can be found in O’Reilly et al. (2016). This model can suitably be integrated into an image-based patient-specific phantom for heterogeneous bone marrow dosimetry during treatment of bone metastases with metastatic castration-resistant prostate cancer (mCRPC) using radiopharmaceuticals emitting alpha-particles. This patient-specific model was used for 3D bone marrow dose calculations in theranostics using ^68^Ga-PSMA-11 and ^177^Lu-PSMA-617 (Gosewisch et al. 2019). This model can be further translated in the course of a clinical mCRPC treatment with ^68^Ga-PSMA-11 and ^225^Ac-PSMA-617 (Kratochwil et al. 2016).

Gersh et al. (2007) have created a simple quadric-based geometric model of trabecular spongiosa designed specifically for implementation into the Monte Carlo radiation transport code PENELOPE. In this study the generation of geometric models of spongiosa is based on spherical bodies. While this model is simple, it has no explicit indication for different tissues within bone. Because the PENELOPE code can only simulate photon and electron transport, this quadric-based geometric model must be constructed in other MC codes like Geant4 or GATE, if applied for radiopharmaceutical therapy using radionuclides emitting alpha particles.

In contrast to the aforementioned path-length and voxel-based trabecular bone model, Hobbs et al. (2012) proposed a simplified cell-level geometric model of a trabecular marrow cavity, which took into account three marrow cell distributions. As shown in Fig. 1C, the model consists of two spherical shells and one inner sphere. The inner sphere indicated in Fig. 1C by a brown circle line represents the deep marrow. The shell between the brown circle line and the bone boundary region (brown shaded area) including the brown speckled ring represents the shallow marrow where the osteoprogenitor cells are present. The shell is 50 μm in depth. The brown speckled shell is 10 μm thick and models the endosteal layer where osteoblasts are present. Altogether, the cavity including the deep marrow (inner sphere), the shallow marrow (middle shell), and the thin endosteal layer constitutes the trabecular marrow cavity and it is surrounded by bone. The hematopoietic stem and progenitor cells are represented by small brown spheres, and the adipose cells are represented by small white spheres. They are present throughout the trabecular marrow cavity. In the MC radiation transport simulation study by Hobbs et al. (2012), sources of ^223^Ra were assumed to be distributed in the endosteal layer or along the trabecular surface. This model took into account different marrow cells. It can be integrated into skeletal targeted dosimetry model for alpha-particle therapy and can provide the heterogeneity of dose distributions on a cellular level, for quantification of any dose–effect relationship.

As the pelvic region, the lumbar spine, the femur and the thoracic spine are regions most commonly affected by bone metastases from prostate cancer, Pinto et al. (2020) constructed a voxel model (in size 6.05 × 6.05 × 6.05 μm) of microstructural trabecular bone using images of a mouse femur obtained with a micro-CT device. A total of 2,080 slices with a square matrix size of 1252 pixels per slice were acquired. Because the structure and the spatial gradient of the radiosensitive cells in the bone marrow cavity of mouse femur is very similar to those of human femurs (Watchman et al. 2007), this image-based microstructural trabecular bone model has been used to investigate the heterogeneous dose distribution within cells (Pinto et al. 2020). The model is suitable for alpha-particle emitting radiopharmaceuticals such as ^225^Ac-PSMA-617 used in therapy.

Tranel et al. (2021) developed a voxel bone marrow model with realistic dimensions. The model includes a voxel size of 10 × 10 × 10 μm^3^. This voxel size corresponds to the size of the cells which are homogenously distributed in the marrow cavity. Furthermore, a blood vessel compartment with a maximum diameter of 50 µm is embedded in the centre of the cylindrical bone marrow model. Radionuclides such as ^211^At and ^225^Ac were located in the blood vessel compartment. The cells which were assumed to be homogenously distributed in the trabecular bone can be considered as tissues at risk, and the absorbed dose in each cell can be calculated. Another unique advantage of this model is that 50 voxels containing radioactivity could be randomly distributed in the trabecular bone compartments. These voxels can be used to model diffusion and infiltration of bone metastases so that the impact of selecting an appropriate radiopharmaceutical can be evaluated.

## Heterogeneity of dose distribution in Xofigo treatment

^223^Ra is a bone seeking radionuclide, it prefers to accumulate in bone and deliver dose to different cells in trabecular bone marrow cavity. Therefore, estimates of the average absorbed dose in bone marrow do not predict the very low haematological toxicity of ^223^Ra dichloride in comparison to other radiopharmaceutical therapy agents (Sgouros et al. 2020; Parker and Sartor 2013). Because of the short range of alpha particles emitted by ^223^Ra and the known localization of this radiotherapeutic agent on the trabecular bone surface, only bone marrow cells within 80 μm of the bone surface are irradiated, meaning that most of the bone marrow space is not irradiated (Hobbs et al. 2012). Trabecular bone marrow models can resolve this discrepancy between the average absorbed dose over bone marrow and the local absorbed dose in trabecular bone marrow by dose calculations that consider the microscale distribution of ^223^Ra within the trabecular bone marrow. In this context one should first look at the heterogeneity of the dose distribution in different organs and tissues. This demonstrates that the dose-limiting organ and tissue in Xofigo therapy are the osteoprogenitor cells within trabecular bone marrow. As introduced in the sub-organ model for bone marrow, the heterogeneity of dose distribution within bone marrow will be presented below. The link of this dose heterogeneity in bone marrow to haematological toxicity is also addressed.

Applying the MIRD/ICRP generalized dose calculation formalism, Höllriegl et al. (2021) calculated organ absorbed doses for a reference population patient by means of biokinetic models of ^223^Ra and its decay products, i.e. ^219^Rn, ^215^Po, ^211^Pb, ^211^Bi, ^211^Po and ^207^Tl developed by ICRP (2017) and the *S* coefficients derived from the ICRP reference male computational voxel phantom (ICRP 2009). Because of the bone-seeking nature and the heterogeneous bio-distribution of ^223^Ra at the organ level, the heterogeneity of doses distributed among organs is obvious. The highest absorbed dose coefficient of 221 mGy MBq^−1^ was found for bone endosteum, followed those for liver, red bone marrow and kidneys (36, 34 and 26 mGy MBq^−1^, respectively). Absorbed dose coefficients to other organs were relatively small. The absorbed organ dose coefficients for endosteal bone surface and red marrow (221 and 34 mGy MBq^−1^, respectively) estimated by Höllriegl et al. (2021) are much lower compared to previous modelling results (750 and 72 mGy MBq^−1^) obtained by Lassmann and Nosske (2013). In contrast, the organ dose coefficient for kidneys (26 mGy MBq^−1^) was much greater in comparison to previous results by Lassmann and Nosske (2013) (3.4 mGy MBq^−1^). Furthermore, the new dose coefficient for colon (5 mGy MBq^−1^) is lower than the previous one (35 mGy MBq^−1^). These differences in organ dose coefficients may partially be explained by the fact that Lassmann and Nosske (2013) used the old biokinetic models of radium and its progeny (ICRP 1993) and *S* coefficients calculated using the previous stylized mathematical phantom (ICRP 1979). For example, the old biokinetic radium model had only one liver compartment and no kidney compartments. Also, the old gastrointestinal tract model (ICRP 1979) included compartments for the upper large intestine and the lower large intestine with no sub-compartments of the colon such as right colon, left colon, and rectosigmoid, which are all included in the new human alimentary tract model (ICRP 2006). As for the observed differences in skeletal dosimetry, the endosteum has recently been defined in the new voxel phantom as a 50 μm thick layer covering the surfaces of the bone trabeculae in regions of trabecular spongiosa and the cortical surfaces of the medullary cavities within the shafts of all long bones (ICRP 2009). In contrast, the bone surfaces in the former model were defined as a single cell layer of 10 μm thickness covering the surfaces of both the bone trabeculae and the Haversian canals of cortical bone (ICRP 1977). Moreover, the new calculation of the skeletal dose coefficients employs improved computational algorithms to estimate the absorbed dose to endosteum and red marrow (ICRP 2016).

Two clinical studies (Chittenden et al. 2015; Yoshida et al. 2016) reported higher absorbed dose coefficients in bone endosteum (5378 and 761 mGy MBq^−1^) and in red bone marrow (408 and 92 mGy MBq^−1^) with a greater discrepancy up to a factor of seven for endosteal bone surface in comparison to modelled results. In the clinical dose assessment, clinical imaging data were evaluated and cumulated activities derived through regions of interest (ROIs). In most cases, the commercial dosimetric software tools will be used. Furthermore, in clinical studies, it is assumed that short-lived progeny deposits directly at the location of its parent radionuclide. This demonstrated the challenges in assessing dose in clinical practice (Flux 2017): the heterogeneous uptake of the ^223^Ra in tissues and organs of patients, the difficulties to correctly determine the region of interests (ROIs) from gamma camera or SPECT images and the quantification of ^223^Ra activities in the organs or tissues and, consequently, the difficulties to estimate the TIA. Furthermore, a high variability of patient anatomy and biokinetics and different clinical techniques for imaging and dose calculations in the different clinics may also contribute to propagation of uncertainties in dose assessments.

The clinically assessed dose coefficients in the bone region mentioned above are much greater than those modelled (Höllriegl et al. 2021; Lassmann and Nosske 2013). In contrast, clinical dose assessment, smaller dose coefficients for kidneys (2 and 6 mGy MBq^−1^) and liver (1.9 and 2 mGy MBq^−1^) were observed in comparison to the modelling results. Finally, the clinical colon dose coefficients (22 and 47 mGy MBq^−1^) are comparable to those modelled (5 and 35 mGy MBq^−1^). The differences mentioned demonstrate the complexity in the clinical dose assessment setting and the recommended biokinetic modelling and dosimetry formalism (Bolch et al. 2009). The clinical studies evaluated clinical imaging data, derived the cumulated activities through ROIs and used the computer software tool OLINDA/EXM (Stabin et al. 2005) for dosimetry, a software based on old dosimetric models and *SAF*s derived from mathematical phantoms (Cristy and Eckerman 1987). Furthermore, in the clinical studies (Chittenden et al. 2015; Yoshida et al. 2016) it was assumed that short-lived progeny deposit energy directly at the location of its parent radionuclide, whereas in the work of Höllriegl et al. (2021) the biokinetics is independently modelled for each progeny separately. This may explain some of the observed differences between modelled and clinical results. It should be also noted that in the calculations, no specific tumour compartment was integrated in the biokinetic models (Höllriegl et al. 2021), which may also be a reason for the differences observed as compared to the clinical dose assessment.

This strongly suggests that in patient dose assessment one should take into consideration not only the heterogeneity in the uptake of radiopharmaceuticals in tissues and organs of individual patients, the determination of ROIs from images used to visualize activity distributions within a patient and estimate of the volume of organs; but also any heterogeneities within the modelling-based dose formalisms. In addition, the dosimetry for radiopharmaceuticals should primarily be assessed based on patient-specific imaging and bio-distribution acquired in the clinical setting. The observed differences point towards large uncertainties and heterogeneities in absorbed dose estimates for the bone marrow that are based on organ-level biokinetic and anatomical models. Consequently, small-scale dosimetry is required to explore the heterogeneity of sub-organ doses and even cellular doses in bone marrow tissue.

Previously, Watchman et al. (2005) performed a simulation of absorbed fractions using the 3D microstructure of the 44 year male bone trabecular and marrow cavities developed by Whitwell and Spiers (1976). As described above, in this 3D model active and inactive tissues within the marrow space are represented, and a 3D chord-length-based transport model (Watchman et al. 2005) was used with defined source and target regions (corresponding to different bone tissues): trabecular bone volume, trabecular bone endosteum, trabecular bone surfaces, trabecular active (“red”) marrow, and trabecular inactive (“yellow”) marrow. By doing so, the absorbed fraction can be evaluated assuming alpha-particle distributions in different source regions in the trabecular bone, and further be used for absorbed dose calculations for active bone marrow. Values of absorbed fraction (for example, 0.81, 0.80, and 0.55 for 6 MeV alpha-particles, and 0.74, 0.72, and 0.43 for 9 MeV alpha-particles (Watchman et al. 2005)) for the self-dose to active bone marrow are considerably lower than 1.0, which is the value assumed in ICRP Publication 30 (ICRP 1979) for the ribs, cervical vertebra, and parietal bone using the ICRP reference marrow cellularity (72%, 72%, and 42%, respectively) for a 25 year adult. Obviously, these AF values will give a lower absorbed dose in red marrow.

AF values (0.95 and 0.28) of marrow cavity (with 100% marrow cellularity) and endosteal layer self-absorption, respectively, for 6 MeV alpha-particles calculated by Hobbs et al. (2012) using a simple geometric model of the cell marrow cavity are comparable to the AF values (0.97 and 0.22, respectively) calculated by Watchman et al. (2005). The dose–cell histograms (Hobbs et al. 2012) show that the heterogeneous distribution of cellular dose depends strongly on the position of the cell within the marrow cavity. The results from this bone marrow cavity model (Hobbs et al. 2012) differ significantly from those calculated with the MIRD scheme which are based on average dose calculation. Assuming 2 Gy as the threshold for possible major hematotoxicity (O’Donoghue et al. 2002), Hobbs et al. (2012) showed that increasing the average absorbed dose to the marrow cavity (for a dose range of 1–20 Gy) results in only a small increase in the number of cells receiving more than 2 Gy) and concluded that dosimetry estimates based on an average absorbed dose to a mixture of bone and marrow tissues (i.e. dosimetry at the organ level) does not reflect the actual biological outcome for radiopharmaceutical therapy with Xofigo.

AF values for endosteal layer self-absorption (0.25 and 0.68) and endosteal layer to active marrow (0.35 and 0.14) calculated by Pinto et al. (2020) assuming ^223^Ra is confined to a 10 µm or a 50 µm endosteal layer, respectively, are comparable to the results by Hobbs et al. (2012) for the 10 µm endosteal layer (0.23 for endosteum self-absorption, and 0.35 endosteum to active marrow). In contrast, the AF values for ^223^Ra distributed in the 50 µm layer (0.68 for endosteum self-absorption and 1.4 for endosteum to active marrow) are greater by a factor of 2.7, and a factor of 2.5 smaller in comparison to the values for the 10 µm endosteal layer. The 50 µm thickness of endosteum was defined by ICRP as the surface of the bone trabeculae in regions of trabecular spongiosa, and it is assumed by ICRP that the endosteum serves as the target tissue for radiogenic bone cancer. This implies that ^223^Ra, which accumulates in the endosteum, delivers greater dose to the endosteum itself in comparison with the dose to the active marrow. For ^223^Ra in the 50 μm thick endosteum, Pinto et al. (2020) calculated the average absorbed doses of 1.7 Gy and 985 mGy for endosteum and the trabecular cavity, respectively.

## Discussion and recommendations

The ultimate goal of radiopharmaceutical therapy is to maximize the tumour response while minimizing adverse toxicity to normal tissue. This drives the research needs in the future, for example for enhancement of dose-driven prescription in combination with activity-based prescription or unique imaging modalities and dosimetry tools to increase the therapeutic index (D_tumour_/D_normal_), where D_tumour_ and D_normal_ represent the doses to the target region (or tumour) and the limiting normal organ or tissues, respectively. In this review, it was first shown that the distribution of radiopharmaceuticals with alpha-particle emitters in normal tissues as an example is heterogeneous, and that it is possible to apply some imaging modalities and mass spectrometry methods for dosimetric purpose. Second, the general internal dosimetry formalism used in nuclear medicine was introduced and the absorbed dose approaches appropriate for radiopharmaceutical therapy were addressed. Third, as example, the dose heterogeneity in bone tissues in case of Xofigo treatment of bone metastases was summarized. Based on this review, in the following several research proposals in the radiation research and radiation dosimetry field related to radiopharmaceutical therapy are recommended, all consistent with the SRAs of MELODI and EURADOS (Harrison et al. 2021; MELODI 2021).

## Bio-distribution of activity

Emission tomographic techniques are typically used to determine bio-distribution at an organ level, but is of limited use for targeted alpha therapy at sub-organ regions, such as bone marrow (Gosewisch et al. 2019). Other complementary methods have been used in various studies to derive radiopharmaceutical distributions at relevant substructures, like bone marrow and endosteum within bone tissues (Pinto et al. 2020). Biokinetic data on a sub-tissue level are needed for developing patient-specific physiologically based pharmacokinetic models. Preclinical studies on animals will continue to play a key role in the investigation of the microscopic distribution of radiopharmaceuticals in animal tissues, to identify potential toxicities for dose-limiting human organs. Pharmacokinetic distributions on sub-organ and smaller levels can certainly be obtained in extracted organs from experimental animals combined with ex vivo high-resolution imaging, for example, with the use of an alpha-camera (Bäck and Jacobsson 2010; Miller 2018). Other imaging techniques, based on mass spectrometry, could be useful for assessing the bio-distribution of radiopharmaceuticals at a microscopic scale. Secondary ion mass spectrometry (SIMS) and coupling between laser ablation and inductively coupled plasma mass spectrometry (LA/ICP-MS) have already been applied for the quantitative imaging of the bio-distribution of long-lived radionuclides in tissue sections of rodents with a resolution down to cellular scale (Tessier et al. 2012; Grijalba et al. 2020). The lack of sensitivity for short-lived radionuclides could be overcome in preclinical studies using analogues long-lived isotopes, for example ^226^Ra for ^223^Ra or ^232^Th for ^227^Th. These techniques could also contribute to study the bio-distribution of the decay products of radiopharmaceuticals that can be separated from their parent due to alpha recoil in targeted alpha therapy.

Preclinical data on activity distributions in animals may then be converted to organ sub-compartments in human whole body (Sgouros et al. 2020). Activity distributions in these sub-compartments can then be integrated over the entire organ contour in patient images which in turn may be obtained with clinical imaging modalities, such as PET/CT and SPECT/CT (Gosewisch et al. 2021). Preclinical studies can therefore provide important information for interpreting radiopharmaceutical therapy outcomes and evaluate the benefit-risk balance of the treatment. General methodology translating animal data to humans can be further explored (NCRP 2015) and applied to sub-tissues, such as bone marrow (Cicone et al. 2020; Beykan et al. 2019).

## Dose distribution

The heterogeneity of ^223^Ra in the bone microenvironment has been demonstrated in studies by means of autoradiography in mice (Abou et al. 2015; Suominen et al. 2017). The heterogeneity of stem cells (hematopoietic stem and progenitor cells) in the endosteum and osteoprogenitor cells in the shallow marrow together with the short ranges of alpha-particles makes bone marrow dosimetry challenging. Furthermore, the dose heterogeneity within a cell and DNA damages play an important role for understanding the key damage processes to bone metastases populated from prostate cancer cells. In the cell, energy deposition due to alpha particles emitted by radiopharmaceuticals used in radiotherapy is of stochastic nature (Gholami et al. 2015). In clinical practice, however, the source geometry used for dose calculations is far from the distribution of ^223^Ra actually present during the treatment of bone metastases with mCRPC. Because of the natural heterogeneity of the ^223^Ra distribution in cellular and subcellular levels, bystander effects need to be investigated to understand the effects of radiopharmaceutical therapy on a tissue level (Rajon et al. 2021; Canter et al. 2021), as well as intracellular signalling and interactions within the microenvironment (Wahl et al. 2021). Other processes such as membrane and mitochondria damage induced by alpha particles may lead to cell death (Fink and Cookson 2005; Pouget and Constanzo 2021). Complex interactions of stem cells and DNA with alpha-particles within the bone marrow microenvironment requires further development of microdosimetry and nanodosimetry models appropriately applied on a cellular and molecular level.

## Impact of heterogeneous dose distribution in bone marrow

Bone marrow is the most critical organ in ^177^Lu-PSMA-ligands therapy. The highly heterogeneous distributions of ^177^Lu within bone tissue makes estimation of red bone marrow doses difficult (Gosewisch et al. 2019). ^225^Ac-PSMA-I&T has been used to treat mCRPC patients with bone metastases after treatment failure of ^177^Lu-PSMA radioligand therapy (RLT) (Zacherl et al. 2021). In both treatment modalities, the dose to bone marrow can be estimated applying mass energy-absorption coefficients on the total bone mixture obtained by MC simulations. Dose volume histograms (DVHs) for bone marrow can estimated with the images simulated by MC results. Bone marrow voxel phantoms based on micro-CT images can be further explored to reduce the impact of dose heterogeneity on the DVH (Tranel et al. 2021; Pinto et al. 2020).

The spatial resolution of SPECT and PET is likely to limit their effectiveness to resolve the influence of heterogeneous microscopic dose distributions on the biological response to radiopharmaceutical therapy. One voxel, which is typically 7–15 mm and 4–6 mm wide for SPECT and PET, respectively, might encompass several tissue substructures possibly differing not only in the actual absorbed dose received, but also in radiosensitivity and functional role within an organ. The exploitation of planar and SPECT imaging in quantifying the activity distribution of radiopharmaceuticals in patient needs further investigations (Lassmann et al. 2021; Sgouros et al. 2021; ICRU 2021).

The purpose of radiopharmaceutical therapy is to optimize treatment and ensure improvement of quality of life for patients. To reach a dosimetry of good quality requires the development of optimized methods and protocols for quantitative imaging, activity quantification, dose calculation, and even radiology, for assessment and improvement of the treatment outcome. To implement such an optimized treatment procedure in clinical practice is challenging (Lassmann et al. 2021). Standard patient dosimetry based on single-time-point acquisition obtained for example from a SPECT scan is used in many clinical centres. Similarly, complementary computational dosimetry is required in radiopharmaceutical therapy taking into account the heterogeneity of radiopharmaceutical distributions in the tissues and tumours in the human body.

## Bioeffect modelling in radiopharmaceutical therapy with alpha-emitters

In radiopharmaceutical therapy, heterogeneity in dose distribution is observed at different spatial scales. From the clinical point of view, biological effects at the tumour tissue and organ level, i.e. tumour control and normal tissue complications, are most important. To quantify these effects, in vivo animal experiments and clinical studies are necessary. These effects, however, can also be estimated based on the dose response at a cellular level (in vitro), and the dose distribution within normal tissue and tumour.

What makes this approach promising is the fact that cell survival fractions can be predicted with reasonable accuracy for heterogeneous dose distributions, if the cell survival of the same cell type upon homogeneous dose distribution is used. Friedrich et al. (2018) studied the effects of focussed spots of ionizing radiation on cells and found that clustering of DNA damage on both nanometre and micrometre scale leads to enhanced cell inactivation compared to a more homogeneous DNA damage distribution. Applying a biophysical model, the local effect model (LEM), they interpreted their observations in terms of enhanced double strand break (DSB) production and DSB interaction, quantitatively decomposing the overall cell killing effects of heterogeneous dose distributions. The LEM I model was already applied in several ion therapy centres to predict the relative biological effectiveness of ions, while the predictive power of the LEM IV model was recently quantified (Pfuhl et al. 2022) using the particle irradiation data set (Friedrich et al. 2012).

The major strength of the LEM is that simulations can be performed completely autonomously without any fitting to measured ion beam effects; it is enough to use photon dose response parameters and experimental parameters (ion type, energy, linear energy transfer, spot beam dimensions, nucleus geometry) as model input (Friedrich et al. 2018). While the LEM is mainly applied for irradiation with ion beams, a LEM-based framework was also used to study experimental gold nanoparticle radiosensitisation data (Lin et al. 2015; Brown and Currell 2017). Considering its effectiveness in predicting cell survival probability, which is a major determinant of tumour control probability and normal tissue complication probability, it is reasonable to assume that the therapeutic outcome of radiopharmaceutical treatment can also be predicted based on dose distributions within normal tissues and tumours using the LEM.

Bystander effects are defined as the induction of biological effects in cells that are not directly traversed by a charged particle but are in proximity to cells that are (Hall and Giaccia 2018). Nagasawa and Little (1992) showed that following a low dose of alpha-particles, a larger proportion of cells showed biological damages than was estimated to have been hit by one alpha-particle; specifically, 30% of the cells showed an increase in sister chromatid exchanges even though less than 1% were calculated to have undergone a nuclear traversal. Bystander effects were mainly investigated using external beam of low and high linear energy transfer radiation (ICRU 2021). However, recent in vivo studies with ^223^Ra dichloride demonstrated radiation-induced bystander effects on disseminated tumour cells in bone marrow (Canter et al. 2021; Rajon et al. 2021). As several emerging alpha-particle emitting radiopharmaceuticals are in clinical trials, such as ^225^Ac-PSMA-617 (NCT04597411) (ClinicalTrials.gov 2022) and ^227^Th conjugate PSMA-TTC (NCT03724747) (Hammer et al. 2020), investigation of the benefit and efficacy of bystander effects for radiopharmaceutical therapy is highly desired.

## Recommendations in view of MELODI and EURADOS strategic research agendas

Integration of heterogeneity at different scales in dose calculations and radiobiological effect modelling is in line with the EURADOS SRA topic “patient dosimetry in nuclear medicine” (Harrison et al. 2021). This topic includes the objectives of the development of cellular dosimetry models together with radiobiological experiments to assess the intracellular activity distribution and relevant biological endpoints; the development of preclinical computational dosimetry to improve the accuracy of dose estimates in preclinical models at the organ and sub-organ level; and the study of dose–effect relationships for internally distributed radionuclides to complement those for external beam radiotherapy. Therefore, further research activities on pharmacokinetics and dosimetry, including small-scale dosimetry and microdosimetry of target molecules, are needed to provide detailed information on the spatial dose distribution as a function of time on organ, sub-organ, cellular, and molecular levels, with emphasis on emerging alpha-particle emitting radiopharmaceuticals.

One of the research priorities of the MELODI SRA is the effects of spatial and temporal variation in radiation dose delivery, which is also gaining importance because of the increasing clinical use of radionuclides (MELODI 2021). Radiopharmaceutical therapy with alpha-emitters typically results in chronic and heterogeneous exposures to both tumours and normal tissues. Since secondary cancers are also of concern, the understanding of the long-term effects due to radiopharmaceutical therapy is of high importance. Understanding the mechanisms involved, especially regarding normal tissues exposed to alpha-particle emitting radionuclides can be an important step towards research related to this priority of MELODI.

Besides the fact that low-dose research can support the development and application of radiopharmaceutical therapy, it can also gain advantages from preclinical and clinical studies on radiopharmaceutical therapy. Considering the effectiveness of mathematical models of cell survival including heterogeneity of energy deposition, it remains an important question how effects at the cellular level manifest themselves at the tissue level. Development of such models and their validation with clinical and preclinical data can be very useful for low-dose research in general, and for the study of effects of spatial variation in dose delivery in particular.

In view of the SRAs of MELODI and EURADOS, several joint research efforts could be performed related to radiopharmaceutical therapy.

1. In cooperation with hospitals and research laboratories, preclinical and clinical data on the heterogeneity of alpha-particle emitting radionuclides should be collected. These data could include imaged-based activity distributions at various scales, such as organ, tissue, cellular, and subcellular scales, as well as blood samples and excretion data. Building up a pharmacokinetic model for specific radiopharmaceuticals, for example, Xofigo and ^177^Lu-PSMA-617, should be feasible. Clinical images for individual patients could be used to set up individual phantoms or images which could further be scaled and integrated to reference phantoms. *S* coefficients could then be calculated for estimating patient organ doses for this radionuclide. Patient-specific biokinetic data at organ and sub-organ levels would facilitate to build individualized pharmacokinetic models.
2. Preclinical and clinical outcomes or radiobiological effects in certain organs or cell lines due to heterogeneous dose distributions resulted by alpha-particle emitting radiopharmaceuticals, such as Xofigo (clinical data) and ^225^Ac-PSMA-I&T (preclinical data) should be reviewed. Dose–response relationship should be investigated from these clinical and preclinical data conducted with dosimetry at different scales.
3. Microdosimetric and nanodosimetric formalisms could be implemented and a quality assurance computation of the microscale *S* coefficients (using the experience about voxel phantoms of organs or tissues, esp. at microscale scale) should be performed for radiopharmaceuticals with alpha-particle emitters. The microscale and nanoscale doses and effects should then be utilized to evaluate specific biologically based quantities i.e. relative biological effectiveness (RBE)-weighted dose, biologically effective dose (BED), equivalent uniform dose (EUD), and other biological models (i.e. LEM and bystander effects) should be exploited to establish a dose–effect relationship for alpha-particle emitting radiopharmaceuticals used in cancer therapy.

## References

[CR1] ClinicalTrials.gov (2022) Study of ^225^Ac-PSMA-617 in men with PSMA-positive prostate cancer. https://clinicaltrials.gov/ct2/show/NCT04597411.

[CR2] Abou DS, Ulmert D, Doucet M, Hobbs RF, Riddle RC, Thorek DLJ (2015). Whole-body and microenvironmental localization of radium-in naïve and mouse models of prostate cancer metastasis. JNCI: J Natl Cancer Inst.

[CR3] Adelstein SJ (1993). Heterogeneity of dose-distribution in nuclear medicine. Proc Twenty-Eight Annu Meet NCRP Radiat Prot Med.

[CR4] Aerts A, Eberlein U, Holm S, Hustinx R, Konijnenberg M, Strigari L, van Leeuwen FWB, Glatting G, Lassmann M (2021). EANM position paper on the role of radiobiology in nuclear medicine. Eur J Nucl Med Mol Imaging.

[CR5] Andreo P (1991). Monte Carlo techniques in medical radiation physics. Phys Med Biol.

[CR6] Attix FH (1986). Introduction to radiological physics and radiation dosimetry.

[CR7] Bäck T, Jacobsson L (2010). The α-camera: a quantitative digital autoradiography technique using a charge-coupled device for ex vivo high-resolution bioimaging of α-particles. J Nucl Med.

[CR8] Beddoe AH, Darley PJ, Spiers FW (1976). Measurements of trabecular bone structure in man. Phys Med Biol.

[CR9] Berger MJ (1973) Improved point kernels for electron and beta-ray dosimetry. Report NBSIR.

[CR10] Beykan S, Fani M, Jensen SB, Nicolas G, Wild D, Kaufmann J, Lassmann M (2019). In vivo biokinetics of ^177^Lu-OPS201 in mice and pigs as a model for predicting human dosimetry. Contrast Media Mol Imaging.

[CR11] Bolch W, Lee C, Wayson M, Johnson P (2010). Hybrid computational phantoms for medical dose reconstruction. Radiat Environ Biophys.

[CR12] Bolch WE, Bouchet LG, Robertson JS, Wessels BW, Siegel JA, Howell RW, Erdi AK, Aydogan B, Costes S, Watson EE, Brill AB, Charkes ND, Fisher DR, Hays MT, Thomas SR (1999). MIRD pamphlet No 17: the dosimetry of non-uniform activity distributions–radionuclide S values at the voxel level. Medical internal radiation dose committee. J Nucl Med off Publ Soc Nucl Med.

[CR13] Bolch WE, Eckerman KF, Sgouros G, Thomas SR (2009). MIRD pamphlet No. 21: a generalized schema for radiopharmaceutical dosimetry-standardization of nomenclature. J Nucl Med.

[CR14] Brown JMC, Currell FJ (2017). A local effect model-based interpolation framework for experimental nanoparticle radiosensitisation data. Cancer Nanotechnol.

[CR15] Canter BS, Leung CN, Fritton JC, Bäck T, Rajon D, Azzam EI, Howell RW (2021). Radium-223–induced bystander effects cause DNA damage and apoptosis in disseminated tumor cells in bone marrow. Mol Cancer Res.

[CR16] Chittenden SJ, Hindorf C, Parker CC, Lewington VJ, Pratt BE, Johnson B, Flux GD (2015). A phase 1, open-label study of the biodistribution, pharmacokinetics, and dosimetry of ^223^Ra-dichloride in patients with hormone-refractory prostate cancer and skeletal metastases. J Nucl Med.

[CR17] Chouin N, Lindegren S, Frost SHL, Jensen H, Albertsson P, Hultborn R, Palm S, Jacobsson L, Bäck T (2013). Ex vivo activity quantification in micrometastases at the cellular scale using the α-camera technique. J Nucl Med.

[CR18] Cicone F, Denoël T, Gnesin S, Riggi N, Irving M, Jakka G, Schaefer N, Viertl D, Coukos G, Prior JO (2020). Preclinical evaluation and dosimetry of [^111^In]CHX-DTPA-scFv78-Fc targeting endosialin/tumor endothelial marker 1 (TEM1). Mol Imaging Biol.

[CR19] Cristy M, Eckerman KF (1987). Specific absorbed fractions of energy at various ages from internal photon sources, Volume 1 Methods.

[CR20] Cross WG (1968). Variation of beta dose attenuation in different media. Phys Med Biol.

[CR21] Dewaraja YK, Frey EC, Sgouros G, Brill AB, Roberson P, Zanzonico PB, Ljungberg M (2012). MIRD pamphlet No. 23 quantitative SPECT for patient-specific 3-dimensional dosimetry in internal radionuclide therapy. J Nucl Med.

[CR22] EANM (2017). Internal Dosimetry Task Force Report: treatment planning for molecular radiotherapy: potential and prospects.

[CR43] EMA (2018) Human medicine European public assessment report (EPAR): Xofigo (radium-223 dichloride). https://www.ema.europa.eu/en/documents/overview/xofigo-epar-medicine-overview_en.pdf

[CR23] Eberlein U, Cremonesi M, Lassmann M (2017). Individualized dosimetry for theranostics: necessary, nice to Have, or counterproductive?. J Nucl Med.

[CR24] EC (2014). Council Directive 2013/59/Euratom of 5 December 2013 laying down basic safety standards for protection against the dangers arising from exposure to ionising radiation, and repealing Directives 89/618/Euratom, 90/641/Euratom, 96/29/Euratom, 97/43/Euratom and 2003/122/Euratom. Off J Eur Union.

[CR25] Fink SL, Cookson BT (2005). Apoptosis, pyroptosis, and necrosis: mechanistic description of dead and dying eukaryotic cells. Infect Immun.

[CR26] Flux GD (2017). Imaging and dosimetry for radium-223: the potential for personalized treatment. Br J Radiol.

[CR27] Friedrich T, Scholz U, ElsäSser T, Durante M, Scholz M (2012). Systematic analysis of RBE and related quantities using a database of cell survival experiments with ion beam irradiation. J Radiat Res.

[CR28] Friedrich T, Ilicic K, Greubel C, Girst S, Reindl J, Sammer M, Schwarz B, Siebenwirth C, Walsh DWM, Schmid TE, Scholz M, Dollinger G (2018). DNA damage interactions on both nanometer and micrometer scale determine overall cellular damage. Sci Rep.

[CR29] Frost SHL, Frayo SL, Miller BW, Orozco JJ, Booth GC, Hylarides MD, Lin Y, Green DJ, Gopal AK, Pagel JM, Bäck TA, Fisher DR, Press OW (2015). Comparative efficacy of ^177^Lu and ^90^Y for Anti-CD20 pretargeted radioimmunotherapy in murine lymphoma xenograft models. PLoS ONE.

[CR30] Gersh JA, Dingfelder M, Toburen LH (2007). Modeling energy deposition in trabecular spongiosa using the Monte Carlo code PENELOPE. Health Phys.

[CR31] Gholami Y, Zhu X, Fulton R, Meikle S, El-Fakhri G, Kuncic Z (2015). Stochastic simulation of radium-223 dichloride therapy at the sub-cellular level. Phys Med Biol.

[CR32] Gosewisch A, Ilhan H, Tattenberg S, Mairani A, Parodi K, Brosch J, Kaiser L, Gildehaus FJ, Todica A, Ziegler S, Bartenstein P, Böning G (2019). 3D Monte Carlo bone marrow dosimetry for Lu-177-PSMA therapy with guidance of non-invasive 3D localization of active bone marrow via Tc-99m-anti-granulocyte antibody SPECT/CT. EJNMMI Res.

[CR33] Gosewisch A, Schleske M, Gildehaus FJ, Berg I, Kaiser L, Brosch J, Bartenstein P, Todica A, Ilhan H, Böning G (2021). Image-based dosimetry for 225Ac-PSMA-I&T therapy using quantitative SPECT. Eur J Nucl Med Mol Imaging.

[CR34] Graves SA, Hobbs RF (2021). Dosimetry for optimized, personalized radiopharmaceutical therapy. Semin Radiat Oncol.

[CR35] Grijalba N, Legrand A, Holler V, Bouvier-Capely C (2020). A novel calibration strategy based on internal standard–spiked gelatine for quantitative bio-imaging by LA-ICP-MS: application to renal localization and quantification of uranium. Anal Bioanal Chem.

[CR36] Hall EJ, Giaccia AJ (2018). Radiobiology for the radiologist.

[CR37] Hammer S, Hagemann UB, Zitzmann-Kolbe S, Larsen A, Ellingsen C, Geraudie S, Grant D, Indrevoll B, Smeets R, von Ahsen O, Kristian A, Lejeune P, Hennekes H, Karlsson J, Bjerke RM, Ryan OB, Cuthbertson AS, Mumberg D (2020). Preclinical efficacy of a PSMA-targeted thorium-227 conjugate (PSMA-TTC), a targeted alpha therapy for prostate cancer. Clin Cancer Res.

[CR38] Harrison RM, Ainsbury E, Alves J, Bottollier-Depois JF, Breustedt B, Caresana M, Clairand I, Fantuzzi E, Fattibene P, Gilvin P, Hupe O, Knežević Ž, Lopez MA, Olko P, Olšovcová V, Rabus H, Rühm W, Silari M, Stolarczyk L, Tanner R, Vanhavere F, Vargas A, Woda C (2021). EURADOS strategic research agenda 2020: vision for the dosimetry of ionising radiation. Radiat Prot Dosimetry.

[CR39] Hobbs RF, Song H, Watchman CJ, Bolch WE, Aksnes AK, Ramdahl T, Flux GD, Sgouros G (2012). A bone marrow toxicity model for ^223^Ra alpha-emitter radiopharmaceutical therapy. Phys Med Biol.

[CR40] Hobbs RF, Baechler S, Fu D-X, Esaias C, Pomper MG, Ambinder RF, Sgouros G (2011). A model of cellular dosimetry for macroscopic tumors in radiopharmaceutical therapy. Med Phys.

[CR41] Höllriegl V, Petoussi-Henss N, Hürkamp K, Ocampo Ramos JC, Li WB (2021). Radiopharmacokinetic modelling and radiation dose assessment of 223Ra used for treatment of metastatic castration-resistant prostate cancer. EJNMMI Phys.

[CR42] Hough M, Johnson P, Rajon D, Jokisch D, Lee C, Bolch W (2011). An image-based skeletal dosimetry model for the ICRP reference adult male–internal electron sources. Phys Med Biol.

[CR44] ICRP (1977). Recommendations of the ICRP. ICRP Publication 26. Ann ICRP.

[CR45] ICRP (1979). Limits for intakes of radionuclides by workers ICRP Publication 30, Part 1. Ann ICRP.

[CR46] ICRP (1993). Age-dependent doses to members of the public from intake of radionuclides-Part 2 Ingestion dose coefficients ICRP Publication 67. Ann ICRP..

[CR47] ICRP (2006). Human alimentary tract model for radiological protection. ICRP publication 100. Ann ICRP.

[CR48] ICRP (2009). Adult reference computational phantoms. ICRP publication 110. Ann ICRP.

[CR49] ICRP (2016). The ICRP computational framework for internal dose assessment for reference adults: specific absorbed fractions. ICRP Publication 133. Ann ICRP.

[CR50] ICRP (2017). Occupational intakes of radionuclides: Part 3. ICRP publication 137. Ann ICRP.

[CR51] ICRP (2019). Radiological protection in therapy with radiopharmaceuticals. ICRP Publication 140. Ann ICRP.

[CR52] ICRU (1979) Methods of assessment of absorbed dose in clinical use of radionuclides. ICRU Report No. 32:1–62. International Commission on Radiation Units and Measurements

[CR53] ICRU (2002). Absorbed-dose specification in nuclear medicine ICRU Report No. 67. J ICRU.

[CR54] ICRU (2021). Dosimetry-guided radiopharmaceutical therapy. CRU Report No. 96. J ICRU.

[CR55] Jokisch DW, Patton PW, Inglis BA, Bouchet LG, Rajon DA, Rifkin J, Bolch WE (1998). NMR microscopy of trabecular bone and its role in skeletal dosimetry. Health Phys.

[CR56] Kielar KN (2009). Bone marrow dosimetry via microCT imaging and stem cell spatial mapping.

[CR57] Konijnenberg M, Herrmann K, Kobe C, Verburg F, Hindorf C, Hustinx R, Lassmann M (2021). EANM position paper on article 56 of the Council Directive 2013/59/Euratom (basic safety standards) for nuclear medicine therapy. Eur J Nucl Med Mol Imaging.

[CR58] Kratochwil C, Bruchertseifer F, Giesel FL, Weis M, Verburg FA, Mottaghy F, Kopka K, Apostolidis C, Haberkorn U, Morgenstern A (2016). ^225^Ac-PSMA-617 for PSMA-targeted α-radiation therapy of metastatic castration-resistant prostate cancer. J Nucl Med.

[CR59] Kunos CA, Mankoff DA, Schultz MK, Graves SA, Pryma DA (2021). Radiopharmaceutical chemistry and drug development—what’s changed?. Semin Radiat Oncol.

[CR60] Lassmann M, Nosske D (2013). Dosimetry of 223Ra-chloride: dose to normal organs and tissues. Eur J Nucl Med Mol Imaging.

[CR61] Lassmann M, Eberlein U, Gear J, Konijnenberg M, Kunikowska J (2021). Dosimetry for radiopharmaceutical therapy: the European perspective. J Nucl Med.

[CR62] Lin Y, McMahon SJ, Paganetti H, Schuemann J (2015). Biological modeling of gold nanoparticle enhanced radiotherapy for proton therapy. Phys Med Biol.

[CR63] Ljungberg M, Sjögreen Gleisner K (2016). Personalized dosimetry for radionuclide therapy using molecular imaging tools. Biomedicines.

[CR64] Ljungberg M, Gleisner KS (2015). Hybrid imaging for patient-specific dosimetry in radionuclide therapy. Diagnostics (basel).

[CR65] Ljungberg M, Sjogreen Gleisner K (2018). 3-D image-based dosimetry in radionuclide therapy. IEEE Transac Radiat Plasma Med Sci.

[CR66] Loevinger R, Budinger T, Watson E (1991). MIRD primer for absorbed dose calculations.

[CR67] Loevinger R, Berman M (1975). MIRD Pamphlet No. 1 revised: a revised schema for calculating the absorbed dose from biologically distributed radionuclides.

[CR68] McDevitt MR, Sgouros G, Finn RD, Humm JL, Jurcic JG, Larson SM, Scheinberg DA (1998). Radioimmunotherapy with alpha-emitting nuclides. Eur J Nucl Med.

[CR69] MELODI (2021) Strategic research agenda of the multidisciplinary European low dose initiative. https://melodi-online.eu/wp-content/uploads/2021/10/MELODI-SRA-2021-FINAL-post-consultation.pdf 10.1007/s00411-017-0726-1 PMC5816101 29247291

[CR70] Miller BW (2018). Radiation imagers for quantitative, single-particle digital autoradiography of alpha- and beta-particle emitters. Semin Nucl Med.

[CR71] Miller BW, Gregory SJ, Fuller ES, Barrett HH, Bradford Barber H, Furenlid LR (2014). The iQID camera: an ionizing-radiation quantum imaging detector. Nucl Instrum Methods Phys Res, Sect A.

[CR72] Miller BW, Frost SHL, Frayo SL, Kenoyer AL, Santos E, Jones JC, Green DJ, Hamlin DK, Wilbur DS, Fisher DR, Orozco JJ, Press OW, Pagel JM, Sandmaier BM (2015). Quantitative single-particle digital autoradiography with α-particle emitters for targeted radionuclide therapy using the iQID camera. Med Phys.

[CR73] MIRD (1968). MIRD pamphlet No. 1: A schema for absorbed-dose calculations for biologically distributed radionuclides. J Nucl Med.

[CR74] Nagasawa H, Little JB (1992). Induction of sister chromatid exchanges by extremely low doses of alpha-particles. Cancer Res.

[CR75] NCRP (2015) Extrapolation of radiation–induced cancer risks from nonhuman experimental systems to humans. NCRP Report No. 150. National Council on Radiation Protection and Measurements,

[CR76] O'Donoghue JA, Baidoo N, Deland D, Welt S, Divgi CR, Sgouros G (2002). Hematologic toxicity in radioimmunotherapy: dose-response relationships for I-131 labeled antibody therapy. Cancer Biother Radiopharm.

[CR77] O'Reilly SE, DeWeese LS, Maynard MR, Rajon DA, Wayson MB, Marshall EL, Bolch WE (2016). An image-based skeletal dosimetry model for the ICRP reference adult female-internal electron sources. Phys Med Biol.

[CR78] Parker C, Sartor O (2013). Radium-223 in prostate cancer. N Engl J Med.

[CR79] Pfuhl T, Friedrich T, Scholz M (2022). Comprehensive comparison of local effect model IV predictions with the particle irradiation data ensemble. Med Phys.

[CR80] Pinto GM, Bonifacio DAB, de Sá LV, Lima LFC, Vieira IF, Lopes RT (2020). A cell-based dosimetry model for radium-223 dichloride therapy using bone micro-CT images and GATE simulations. Phys Med Biol.

[CR81] Pouget J-P, Constanzo J (2021). Revisiting the radiobiology of targeted alpha therapy. Front Med.

[CR82] Rajon DA, Canter BS, Leung CN, Bäck TA, Fritton JC, Azzam EI, Howell RW (2021). Modeling bystander effects that cause growth delay of breast cancer xenografts in bone marrow of mice treated with radium-223. Int J Radiat Biol.

[CR83] Sgouros G, Hobbs RF (2014). Dosimetry for radiopharmaceutical therapy. Semin Nucl Med.

[CR84] Sgouros G, Frey E, Wahl R, He B, Prideaux A, Hobbs R (2008). Three-dimensional imaging-based radiobiological dosimetry. Semin Nucl Med.

[CR85] Sgouros G, Bodei L, McDevitt MR, Nedrow JR (2020). Radiopharmaceutical therapy in cancer: clinical advances and challenges. Nat Rev Drug Discovery.

[CR86] Sgouros G, Frey E, Du Y, Hobbs R, Bolch W (2021). Imaging and dosimetry for alpha-particle emitter radiopharmaceutical therapy: improving radiopharmaceutical therapy by looking into the black box. Eur J Nucl Med Mol Imaging.

[CR87] Sgouros G, Roeske JC, McDevitt MR, Palm S, Allen BJ, Fisher DR, Brill AB, Song H, Howell RW, Akabani G, Committee SM, Bolch WE, Brill AB, Fisher DR, Howell RW, Meredith RF, Sgouros G, Wessels BW, Zanzonico PB (2010). MIRD Pamphlet No. 22 (abridged): radiobiology and dosimetry of alpha-particle emitters for targeted radionuclide therapy. J Nucl Med.

[CR88] Shah A (2004). Reference skeletal dosimetry model for an adult male radionuclide therapy patient based on 3D imaging and paired-image radiation transport.

[CR89] St James S, Bednarz B, Benedict S, Buchsbaum JC, Dewaraja Y, Frey E, Hobbs R, Grudzinski J, Roncali E, Sgouros G, Capala J, Xiao Y (2021). Current status of radiopharmaceutical therapy. Int J Radiat Oncol Biol Phys.

[CR90] Stabin M, Xu XG (2014). Basic principles in the radiation dosimetry of nuclear medicine. Semin Nucl Med.

[CR91] Stabin MG, Sparks RB, Crowe E (2005). OLINDA/EXM: the second-generation personal computer software for internal dose assessment in nuclear medicine. J Nucl Med.

[CR92] Stokke C, Gabiña PM, Solný P, Cicone F, Sandström M, Gleisner KS, Chiesa C, Spezi E, Paphiti M, Konijnenberg M, Aldridge M, Tipping J, Wissmeyer M, Brans B, Bacher K, Kobe C, Flux G (2017). Dosimetry-based treatment planning for molecular radiotherapy: a summary of the 2017 report from the internal dosimetry task force. EJNMMI Phys.

[CR93] Suominen MI, Fagerlund KM, Rissanen JP, Konkol YM, Morko JP, Peng Z, Alhoniemi EJ, Laine SK, Corey E, Mumberg D, Ziegelbauer K, Käkönen SM, Halleen JM, Vessella RL, Scholz A (2017). Radium-223 inhibits osseous prostate cancer growth by dual targeting of cancer cells and bone microenvironment in mouse models. Clin Cancer Res.

[CR94] Tessier C, Suhard D, Rebière F, Souidi M, Dublineau I, Agarande M (2012). Uranium microdistribution in renal cortex of rats after chronic exposure: a study by secondary ion mass spectrometry microscopy. Microsc Microanal.

[CR95] Tranel J, Feng FY, James SS, Hope TA (2021). Effect of microdistribution of alpha and beta-emitters in targeted radionuclide therapies on delivered absorbed dose in a GATE model of bone marrow. Phys Med Biol.

[CR96] Wahl RL, Sgouros G, Iravani A, Jacene H, Pryma D, Saboury B, Capala J, Graves SA (2021). Normal-tissue tolerance to radiopharmaceutical therapies, the knowns and the unknowns. J Nucl Med.

[CR97] Watchman CJ, Jokisch DW, Patton PW, Rajon DA, Sgouros G, Bolch WE (2005). Absorbed fractions for alpha-particles in tissues of trabecular bone: considerations of marrow cellularity within the ICRP reference male. J Nucl Med.

[CR98] Watchman CJ, Bourke VA, Lyon JR, Knowlton AE, Butler SL, Grier DD, Wingard JR, Braylan RC, Bolch WE (2007). Spatial distribution of blood vessels and CD34+ hematopoietic stem and progenitor cells within the marrow cavities of human cancellous bone. J Nucl Med.

[CR99] Whitwell JR (1973). Theoretical investigations of energy loss by ionizing particles in bone.

[CR100] Whitwell JR, Spiers FW (1976). Calculated beta-ray dose factors for trabecular bone. Phys Med Biol.

[CR101] Xu XG, Eckerman KF (2009). Handbook of anatomical models for radiation dosimetry.

[CR102] Yoshida K, Kaneta T, Takano S, Sugiura M, Kawano T, Hino A, Yamamoto T, Shizukuishi K, Kaneko M, Zurth C, Inoue T (2016). Pharmacokinetics of single dose radium-223 dichloride (BAY 88–8223) in Japanese patients with castration-resistant prostate cancer and bone metastases. Ann Nucl Med.

[CR103] Zacherl MJ, Gildehaus FJ, Mittlmeier L, Böning G, Gosewisch A, Wenter V, Unterrainer M, Schmidt-Hegemann N, Belka C, Kretschmer A, Casuscelli J, Stief CG, Unterrainer M, Bartenstein P, Todica A, Ilhan H (2021). First clinical results for PSMA-targeted α-therapy using ^225^Ac-PSMA-I&T in advanced-mCRPC patients. J Nucl Med.

